# Feasibility and acceptability of using biometric fingerprinting to track migrations and support retention in HIV prevention research in fishing population in East Africa

**DOI:** 10.1186/s12889-023-17339-3

**Published:** 2023-12-01

**Authors:** Andrew Abaasa, Paul Mee, Agnes Nanyonjo, Sue Easton, Frank Tanser, Gershim Asiki

**Affiliations:** 1 MRC/UVRI and LSHTM Uganda Research Unit, Entebbe, Uganda; 2 Department of Infectious Disease Epidemiology, London School of Hygiene and Tropical Medicine, London, UK; 3 Lincoln International Institute for Rural Health, College of Health and Science, University of Lincoln, Lincoln, UK; 4 Centre for Epidemic Response and Innovation (CERI), School of Data Science and Computational Thinking, Stellenbosch University, Stellenbosch, South Africa; 5 Department of Chronic Diseases Management Unit, Health and Well-being Theme, African Population and Health Research Center, Nairobi, Kenya

**Keywords:** Biometric fingerprinting fishing migrations track HIV prevention research

## Abstract

**Introduction:**

Fishing populations constitute a suitable key population amongst which to conduct HIV prevention trials due to very high HIV prevalence and incidence, however, these are highly mobile populations. We determined the feasibility and acceptability of using fingerprinting and geographical positioning systems to describe mobility patterns and retention among fisherfolks on the shoreline of Lake Victoria in South-western Uganda.

**Methods:**

Between August 2015 and January 2017, two serial cross-sectional surveys were conducted during which fingerprinting of all residents aged 18–30 years on the shoreline of Lake Victoria was done. A mapper moving ahead of the survey team, produced village maps and took coordinates of every household. These were accessed by the survey team that assigned household and individual unique identifiers (ID) and collected demographic data. Using the assigned IDs, individuals were enrolled and their fingerprints scanned. The fingerprinting was repeated 6 months later in order to determine the participant’s current household. If it was different from that at baseline, a new household ID was assigned which was used to map migrations both within and between villages.

**Results:**

At both rounds, over 99% accepted to be fingerprinted. No fingerprinting faults were recorded at baseline and the level was under 1% at round two. Over 80% of the participants were seen at round two and of these, 16.3%, had moved to a new location whilst the majority, 85%, stayed within the same village. Movements between villages were mainly observed for those resident in large villages. Those who did not consider a fishing village to be their permanent home were less likely to be migrants than permanent residents (adjusted odds ratio = 0.37, 95%CI:0.15–0.94).

**Conclusion:**

Use of fingerprinting in fishing populations is feasible and acceptable. It is possible to track this mobile population for clinical trials or health services using this technology since most movements could be traced within and between villages.

## Introduction

The HIV epidemic continues to be a global challenge and Sub-Saharan Africa is disproportionately affected [1]. The majority of the new HIV infections continue to occur in this region [1]. The new infections are occurring in the presence of a number of proven HIV prevention interventions such as voluntary medical male circumcision and pre-exposure prophylaxis. The continued need to search for more effective interventions to complement the existing efforts including the development of an HIV preventive vaccine are necessary in order to achieve the UNAIDS 95:95:95 targets for HIV elimination [2, 3]. HIV vaccines currently being developed need to be tested in efficacy trials. One of the critical elements of an HIV vaccine efficacy trial is the identification of populations with high rates of new HIV infections who are willing to participate and who can be efficiently recruited and retained for the duration of the vaccine trial [4–6]. Fisherfolk (fishermen and those in fishing related services on the Lake shore) on the shoreline of Lake Victoria have been identified as a population with the potential for inclusion in future HIV vaccine efficacy trials because of their high HIV prevalence > 20% [7, 8] and incidence of more than 3/100 person years at risk [4, 9–11], willingness to participate > 90% [12] and reasonable levels of retention in follow up (> 70%) [13].

Mobility in these communities is however a major challenge in access to health services or participating in clinical trials. Therefore, new strategies for retention are required to achieve the desired high levels of retention for efficacy trials. In the International AIDS Vaccine Initiative (IAVI) cohorts and other studies in the fishing communities [13, 14], phone calls and home visits to remind volunteers to attend follow up clinic visits have been used to enhance retention. Yet despite this effort, the retention in these communities has remained at approximately 75% for all ages and as low as 70% in those aged 18–24 years [13]. Individuals in the fishing communities move from one community to another depending on fish catch levels. Tracking them by home visits or phone calls is challenging because they often move to places with no access to phone networks. During studies in which locator information is continuously updated of any changes such as phone numbers and physical locations, many changes are observed. The high level of mobility, combined with limited phone connectivity calls for the exploration of alternative cost-effective strategies for recruiting, retaining and identifying this population.

Biometric Identification Systems (BIS) have been used for over 100 years in medical and legal inquiries [15]. Mobile BIS technologies have advanced to such a level that portable scanners and adequate power backup systems are available for field use. Iris and fingerprint recognition technologies have been reported to have high accuracy rates, and are useful in matching records in large relational databases [15]. This technology has previously been used successfully in studies such as the SonLa study for identification of clinical trial participants in Vietnam [16], tracking mobile pastoralists in Chad [17] in Sub-Saharan Africa, and for linking health facility data with demographic surveillance data in South Africa [18]. In this study, we used BIS technology for tracking movements within and across fishing communities. Here, we present the findings on the feasibility and acceptability of the finger print technology and describe the movement patterns of the fisherfolk.

## Methods

### Design

Two serial cross-sectional surveys consisting of demographic surveillance and fingerprinting were completed between August 2015 and January 2017. The first (baseline “census”) survey was conducted between August 2015 and February 2016, and round 2 from July 2016 to January 2017.

### Population and location

**Fig. 1:**
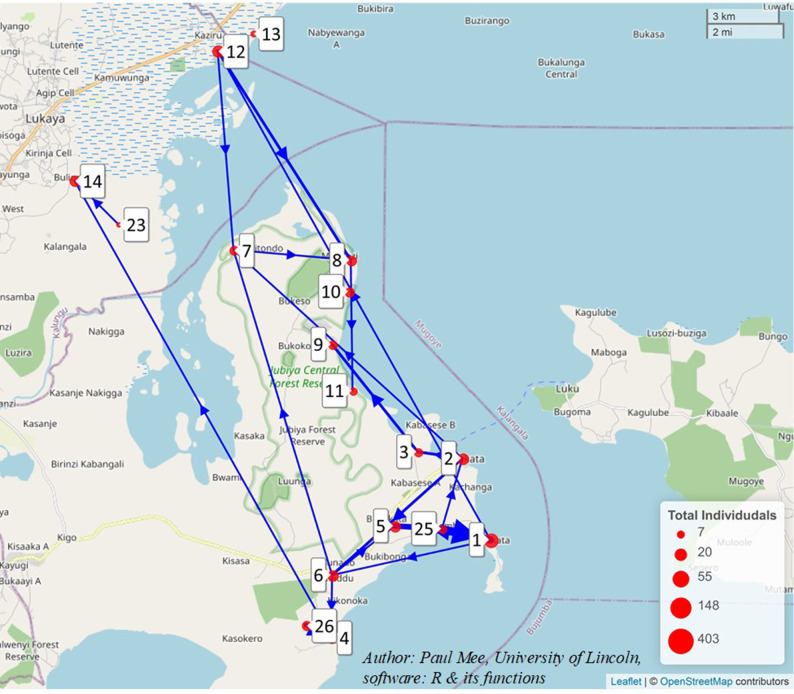
Map showing the location of the fishing communities in the study

The study was conducted in 18 fishing villages, Fig. 1 below (coded sequentially) with an estimated total population of 8,000 adults in 4000 households in Masaka and Kalungu districts (unpublished data from census supported by IAVI in May 2013). Convenience sampling was used to include villages that had a baseline population size of at least 50 people and structures of a typical fishing village (estimates provided in the IAVI Census of 2013). The target study population were those aged between 18 and 30 years as our previous studies had shown this age group to be the most mobile.

### Recruitment

A trained mapper moving ahead of the survey team provided an identification (ID) number to every household (coded from 1 to the last available number) stratified by all participating fishing villages. For every household, the mapper wrote the ID on the household main door and recorded the geographical position system (GPS) coordinates and the household identification number. The mapper further drew a village map to facilitate/guide the movement of the survey team. The survey team used the village map and household numbers to complete the surveys. In the initial round (round one), the survey team provided every household member with a unique identifier in form of a combination of fishing village number, household number and individual’s number. The team completed a census using a handheld computer linked by internet to a central database hosted on a server at MRC/UVRI & LSHTM Uganda research Unit field station in Masaka City, about 40 km from the fishing villages. An offline mode of the database was also available in case of poor internet connectivity.

A standard questionnaire was used to collect data on personal characteristics such as age, sex, length of residence in the current place (census status), current and past marital status, educational level, tribe, religion and position in household (relationships). Due to the high level of mobility of these communities, a module on residence and mobility was included, with questions on: whether this was the participant’s home, usual number of nights away (e.g. for work), usual residence in this household during the last week and previous year, current primary fishing site and future residential village if a move was planned.

### Fingerprinting

Upon completion of the census, the survey team obtained consent for enrolment into a fingerprinting exercise for household members aged 18–30 years. Those consenting had a field fingerprint reader ((TFT 500P VX 10.0 manufacturer Grinding technology co., ltd, China) scan and record the left-hand thumb print at enrolment. The machine automatically assigned a unique identification number, date and time and provided a space for entry of the study identification number (assigned during the census). The survey team entered the household identifier earlier assigned in the census for ease of future data matching. Where the left-hand thumbprint was damaged or could not be recorded, a right-hand print was used. Two fingerprinting machines that could store up to 10,000 unique prints were used for this exercise with data collected on a daily basis. A computer on a local area network acting as a server had a software (Comm Master version 1.43) installed at the MRC/UVRI & LSHTM site which was used to download and manage the fingerprint templates. In a bid to have similar fingerprint data on each scanner at all times, data from both machines was merged and uploaded to both machines using an external flash drive on a daily basis. Backups were done before and after merging to avoid possible loss. At round two the census and fingerprinting exercise was repeated, with the same eligibility criteria as in round one. The participant’s left-hand thumb was scanned and the system searched the database on the machine for a matching fingerprint. If the record already existed, the system would prompt the field-worker showing that the participant was already in the system and display their identification number (ID). If the participant was still in the same village and household as at round one, it was noted in the machine. If the participant had relocated from another fishing village or household or came from outside the fishing villages, a new ID was assigned at the current location using same format. This person/participant ID was used to map migration both within and between villages. At each fingerprinting cycle, any reason for fingerprint failure such as; refusal, finger amputations or injuries, fingerprint reader or computer system malfunctioning due to hardware or software failures etc. were recorded to help improve future use of this technology.

### Statistical analysis

Data collected at both rounds was managed in a Microsoft Access database (Microsoft Corporation, Redmond, WA, 2003), cleaned and analysed in STATA version 15.0 (Stata Corp, College Station, TX, USA). For confidentiality purposes, fishing communities were identified using identification code numbers instead of names. Relatedly, to avoid participants and others identifying their place of residence, where there were less than 4 participants moving to another location, these were reported as ‘less than 4’ instead of the actual number. The characteristics of participants aged 18–30 years were summarized using frequencies and percentages and stratified by sex. To answer the feasibility and acceptability questions, we estimated proportions of participants accepting fingerprinting at both rounds & numbers of machine failures. To answer the primary objectives; mobility of fisherfolk, we summarized the number completing each fingerprinting round, those that moved from the baseline position, using frequencies and percentages. We further examined whether participants’ characteristics were associated with mobility by fitting binomial logistic regression models with 95% confidence intervals (CI). We developed both bivariable and multivariable models. In the bivariable analysis, factors that showed a p-value < 0.20 for likelihood ratio test (LRT) were included in the multivariable analysis. Sex, age group and time away from the fishing village that were included based on a priori hypotheses about their association with the outcome. Factors were removed from the multivariable model using a backward elimination algorithm if removing a factor did not make the fit of the model significantly worse at the 5% level on a likelihood ratio test. The map of migration patterns was developed using the freely available R Statistical Software v4.2.3 [19] (Fig. 1). Specifically the R packages leaflet [20] and leaflegend [21] and the addFlows function in the R package leaflet.minicharts [22] were used.

## Results

Table 1, shows the characteristics of the participants’ fingerprinted at enrolment. Of those aged 18–30 years, a higher proportion of females (53.9%) than males (46.1%) were available for fingerprinting. Of these, about two-thirds were of Baganda tribe, the indigenous occupants of the shoreline of Lake Victoria while the rest were migrants into these fishing villages. About 90% of both males and females, considered the fishing villages as their permanent home. A slightly higher percentage of males (21.1%) than females (5.6%) spend at least a day in a typical week outside their home fishing village. Most of these movements (moves each week) were to another fishing village, 76.6% for males vs. 51.6% for females, Table 1.

**Table 1:** Characteristics of participants available for fingerprinting in round one (age 18–30 years) n = 2,158

		Male (n = 995)		Female (n = 1,163)
Variable	Sub category	n (%)		n (%)
Age group (years)	18–24	461 (46.3)		623 (53.6)
	25–30	534 (53.7)		540 (46.4)
Tribe	Baganda	654 (65.7)		711 (61.1)
	Banyankole	96 (9.7)		171 (14.7)
	Banyarwanda	82 (8.2)		114 (9.8)
	Ugandan Rwandese	45 (4.5)		60 (5.2)
	Other	118 (11.9)		107 (9.2)
Religion	Christian	800 (80.4)		916 (78.8)
	Muslim	195 (19.6)		247 (21.2)
Current location permanent home	Yes	879 (88.3)		1082 (93.0)
	No	116 (11.7)		81 (7.0)
Typical week time (days) spent away	None	785 (78.9)		1098 (94.4)
	1–6	141 (14.2)		21 (1.8)
	> 6	69 (6.9)		44 (3.8)
Where do you normally go to	Another FV	157 (76.6)		32 (51.6)
	Home village outside FV	48 (23.4)		30 (48.4)
Fishing village (FV) identification number	1	239 (24.0)		272 (23.4)
	2	47 (4.7)		87 (7.5)
	3	20 (2.0)		28 (2.4)
	4	91 (9.2)		114 (2.4)
	5	56 (5.7)		60 (5.2)
	6	73 (7.4)		89 (7.7)
	7	36 (3.6)		31 (2.7)
	8	62 (6.2)		63 (5.5)
	9	22 (2.2)		22 (1.9)
	10	40 (4.0)		25 (2.1)
	11	13 (1.3)		18 (1.6)
	12	118 (11.9)		142 (12.2)
	13	9 (0.9)		7 (0.6)
	14	49 (4.9)		74 (6.4)
	23	4 (0.4)		7 (0.6)
	25	55 (5.5)		68 (5.9)
	26	35 (3.5)		32 (2.8)
	27	26 (2.6)		24 (2.1)

*FV-Fishing village*

### Migrations tracked by fingerprints

Over the two fingerprinting rounds, over 80% completed the fingerprinting exercise, with the highest percentage (93.6%) of the participants doing so at round one, Table 2.

**Table 2:** Number fingerprinted and reason for not finger printing

Survey round	Yes	No	Total	Reason for not fingerprinting	Number
One	2020	138	2,158	Away, within fishing Village	56
	93.6%	6.4%	100%	Away, outside fishing village	70
				Willing, machine failure	0
				Finger injured	0
				Declined	12
Two	1687	400	2078	Away, within fishing Village	38
	80.8%	19.2%	100%	Away, outside fishing village	305
				Willing, machine failure	33
				Finger injured	14
				Declined	10
Total	3707	538	4245		
	87.3%	12.7%	100%		

In both rounds, the primary reason for there being no fingerprint recorded among those eligible was having relocated their permanent residence either to another fishing village or another location outside the fishing villages. There were only 22 individuals who refused to participate across both rounds. At round one, two participants were found in two different places within a fishing village before completion of this round. During round two, 275 of 1687 (16.3%) of those fingerprinted had moved from their first households: 40 moving to other fishing villages and 235 moving within the same village, (Table 3)

**Table 3:** Numbers of migrations within the study population between round 1 and round 2 where migrations occur, within village migrations are shown in bold

		Destination Village																
Origin Village	Pop (R1)	1	2	3	4	5	6	7	8	9	11	12	13	14	25	26	27	Total Migrations
**1**	**526**	**85**	0	0	0	< 4	< 4	0	0	0	0	< 4	0	0	0	0	0	**89**
**2**	**137**	0	**12**	< 4	0	0	< 4	< 4	0	0	0	0	0	0	0	0	0	**17**
**3**	**44**	0	0	**8**	0	0	0	0	0	< 4	0	0	0	0	0	0	0	**10**
**4**	**143**	0	0	0	**20**	0	0	0	0	0	0	0	0	< 4	0	0	0	**21**
**5**	**114**	0	0	0	0	**12**	0	0	0	0	0	0	0	0	< 4	0	0	**14**
**6**	**117**	0	0	0	< 4	< 4	**11**	< 4	0	0	0	0	0	0	0	0	0	**14**
**7**	**62**	0	0	0	0	0	0	**8**	< 4	0	0	0	0	0	0	0	0	**9**
**8**	**89**	0	0	0	0	0	0	0	**20**	0	< 4	0	0	0	0	0	0	**21**
**9**	**47**	0	0	0	0	0	0	0	0	**0**	0	0	0	0	0	0	0	**0**
**11**	**29**	0	0	0	0	0	0	0	0	0	**9**	0	0	0	0	0	0	**9**
**12**	**204**	0	0	0	0	0	0	< 4	< 4	0	0	**18**	0	0	0	0	0	**21**
**13**	**16**	0	0	0	0	0	0	0	0	0	0	0	**5**	0	0	0	0	**2**
**14**	**119**	0	0	0	0	0	0	0	0	0	0	0	0	**8**	0	0	0	**8**
**23**	**8**	0	0	0	0	0	0	0	0	0	0	0	0	< 4	0	0	0	**1**
**25**	**38**	7	< 4	0	0	5	0	0	0	0	0	0	0	0	**11**	0	0	**24**
**26**	**67**	0	0	0	4	0	0	0	0	0	0	0	0	0	0	**4**	0	**8**
**27**	**51**	0	0	0	0	0	0	0	0	0	0	0	0	0	0	0	**7**	**7**
	**Total Migrations**	**92**	**13**	**10**	**25**	**20**	**14**	**11**	**23**	**2**	**10**	**19**	**2**	**10**	**13**	**4**	**7**	

R1-fingerprinting round one, pop-population

Those who did not consider a fishing village to be their permanent home were less likely to be migrants than permanent residents (adjusted odds ratio = 0.37, 95%CI:0.15–0.94) (Table 4).

**Table 4:** Proportions, unadjusted and adjusted factors associated with moving to different location at round two compared to the baseline

Variable	Category	N (%)	Moved N = 275	uOR (95%CI)	LRT-p-value	aOR (95%CI)
Overall		1709 (100)	275 (16.1)			
Sex	Male	694 (40.6)	121 (17.4)	1.0	0.213	1.0
	Female	1,015 (59.4)	154 (15.2)	0.85 (0.65–1.10)		0.81 (0.62–1.06)
Age group	18–24	885 (51.8)	143 (16.2)	1.00	0.938	1.0
	25–30	824 (48.2)	132 (16.1)	0.99 (0.76–1.28)		0.98 (0.76–1.28)
Population size	< 1000	1,107 (64.8)	168 (15.2)	1.0	0.165	1.0
	1000+	602 (35.2)	107 (17.8)	1.21 (0.93–1.58)		1.22 (0.93–1.59)
Tribe	Baganda	1,070 (62.6)	179 (16.7)	1.0	0.403	
	Banyankole	236 (13.8)	30 (12.7)	0.72 (0.48-2.00)		
	Banyarwanda	229 (13.4)	35 (15.3)	0.90 (0.61–1.33)		
	Other	174 (10.2)	31 (17.8)	1.08 (0.71–1.64)		
Religion	Christian	1,345 (78.7)	220 (16.4)	1.0	0.563	
	Muslim	364 (22.3)	55 (15.1)	0.91 (0.66–1.25)		
Consider fishing village permanent home	Yes	1,638 (95.8)	270 (16.5)	1.0	0.020	1.0
	No	71 (4.2)	5 (7.0)	0.38 (0.15–0.96)		0.37 (0.15–0.94)
Time away (days/week)	None	1,514 (88.6)	247 (16.3)	1.0	0.479	1.0
	1–7	195 (11.4)	28 (14.4)	0.86 (0.56–1.31)		0.86 (0.55–1.29)

*uOR-Unadjusted odds ratio, aOR-Adjusted odds ratio, LRT-Likelihood ratio test, CI-Confidence interval*

The location of the fishing communities in the study are shown below.

In this figure, the blue arrows show the direction of the migrations and the line thickness represent the number of migrants moving between a pair of villages. The red circles are scaled to represent the number of individuals surveyed in each village. The labels represent the village codes as used in this study. The figure indicates that the majority of migrations were between the largest villages.

### Migration information from proxy/neighbour comments

Among the people who had left a given fishing village completely, most (15) had left for Kampala, Uganda’s capital and most populous city about 100 km to the North. A further 7 had left for the “islands” of which there are many in the northern part of Lake Victoria. Other destinations included Mbarara City about 150 km inland to the West of this area and the nearby city of Masaka about 15 km away. A few had crossed the border into Tanzania about 150 km Southwest of this area or further afield. The others were recorded as having relocated to other villages or districts.

Among those who were absent temporarily, 28 were in the Islands/Ssese Islands/Kalangala/landing site, and a further 25 on “the Lake”. These, it is implied, were away working at fishing. A further 42 were described simply as “away at work”, so probably also fishing. Of those remaining: 10 were visiting Masaka (city), 11 in Lambu, one of the larger local villages, 5 in Kampala, and 3 in Mbarara. Some absences were described by proxies through activity rather than location. Among these were 20 who were at (boarding) school, 9 who had gone to hospital for treatment, 5 had gone to work in “forestry”, at least 4 in farming, and several were described as working as drivers, boda riders (local motorbike-taxi) or selling fuel. Several were away “looking for work”. The migration for alternative work underlines the uncertainty of income among the fishing community.

## Discussion

In this study, we aimed to determine the feasibility and acceptability of using fingerprint technology in fishing communities to detect mobility and retain fisherfolk in follow up. We found that the fingerprinting technology was feasible and acceptable in these communities. At the initial round almost all eligible participants accepted being fingerprinted and less than 1% declined at the second round. There were isolated incidences where fingerprinting was not feasible due to machine failure, participant declining or having a damaged finger. The results on mobility showed that one in six fisherfolk had moved from their baseline (initial) household position and most of these (85%) moved to another household within the same fishing village. While it is not known why fisherfolks might move from one household to another within the same village, it might be that these movements are associated with evading paying rent or domestic violence [23]. Alternatively, movements to another fishing village in such a short time have been associated with search for better fish catch [24, 25] or farming in bigger villages with adjacent farm land in seasons of low fish catch [26]. We also note that some fisherfolks are temporarily away from their homes as described by proxies; they are in Islands, other landing sites or on the lake fishing. There are those whose work was described as drivers, boda riders (local motorbike-taxi) or selling fuel. These are likely to return to the fishing village of origin. Those described as looking for work in Masaka, Kampala and Mbarara, cities a long distance away, are less likely to return. There were no specific participant characteristics statistically associated with movement to another location from the baseline except for those that did not consider the fishing village their permanent home. Participants not considering fishing villages as their permanent home may not move within or across villages probably because of lack of networks for work in the fishing communities and are likely to be temporary visitors. Though not identified as a factor linked to movement within and between sites, sex work might influence sex workers to move when their clients move. However, not many women identify themselves as sex workers in these communities. Generally, the retention was good, over 80% and suitable for HIV prevention trials. The results of the movement pattern of fisherfolk show that much of the movement is into and between the large villages. Because of resource limitations, HIV prevention and healthcare services and other social amenities such as schools can be centred in the large villages to serve the entire fishing communities. This information can also be used to locate facilities providing health services such as HIV testing, and provision of ART for these communities.

The strengths of this study included, establishment of the village maps, taking of every household coordinates and conducting a census. These were instrumental in enabling fieldworkers to account for every member of all the studied fishing villages. The fingerprinting machines used had very minimal failure rates and could work for long hours before running down the batteries. The study is not without limitations; we studied about 50% of the fishing villages on Lake Victoria shoreline in Masaka district due to funding constraints and therefore could not establish where some of the participants not seen at round two had moved. It is possible that they moved to adjacent villages not included in the study. We collected data at two rounds and therefore missed the opportunity to determine whether those that move ever return to the baseline village and/or household. We included only participants aged 18–30 who are thought to be highly mobile but were found at home at study rounds, not those away fishing. This could introduce a bias linked to obtaining results only generalizable to those likely to be available at home.

## Conclusion

In conclusion, the use of fingerprinting technology is feasible and acceptable in the fishing villages on the shoreline of Lake Victoria. The study was able to demonstrate that fisherfolk mainly move from one household to another within the same fishing village. While there were a lot of movement between the large villages, small villages also attract from and donate to one another probably due to seasonality in fish catch. The fingerprinting technology together with mapping and recording of village and/or household geographical position system coordinates can be helpful in following up fisherfolk and improving the delivery of HIV care to this vulnerable population. A lot of individuals apparently reported as lost to follow up, many have actually migrated within or across fishing villages and could be identified through the use of biometric fingerprinting. This can be a useful resource in retaining in follow up participants enrolling in future HIV prevention trials in these villages and those seeking healthcare services. This technology can also be used to prevent co-enrolments among migratory populations.

## Data Availability

The MRC/UVRI & LSHTM Uganda Research Unit encourages data sharing and has a published data sharing policy (https://web.archive.org/web/20201201165922/http://www.mrcuganda.org/sites/default/files/publications/MRC_UVRI_Data_sharing_policy_December2015.pdf). This policy summarizes the conditions under which data collected by the Unit can be made available to other bona fide researchers, the way in which such researchers can apply to have access to the data and how data will be made available if an application for data sharing is approved. Should any of the other researchers need to have access to the data from which this manuscript was generated, the processes to access the data are well laid out in the policy. The corresponding author can be contacted for any clarifications and/or support to access the data.

## Abbreviations

AIDS: Acquired Immunodeficiency Syndrome
BIS: Biometric Identification System
CI: Confidence Interval
GPS: Geographical Positioning Systems
IAVI: International AIDS Vaccine Initiative
LRT: Likelihood Ratio Test
LSHTM: London School of Hygiene and Tropical Medicine
MRC: Medical Research Council
TX: Texas
USA: United States of America
UNAIDS: Joint United Nations Programmes on HIV/AIDS
UVRI: Uganda Virus Research Institute
WA: Washington
